# Molecular and Cytogenetic Characterization of Six Wheat-*Aegilops markgrafii* Disomic Addition Lines and Their Resistance to Rusts and Powdery Mildew

**DOI:** 10.3389/fpls.2018.01616

**Published:** 2018-11-08

**Authors:** Zhixia Niu, Shiaoman Chao, Xiwen Cai, Rebecca B. Whetten, Matthew Breiland, Christina Cowger, Xianming Chen, Bernd Friebe, Bikram S. Gill, Jack B. Rasmussen, Daryl L. Klindworth, Steven S. Xu

**Affiliations:** ^1^Cereal Crops Research Unit, Red River Valley Agricultural Research Center, United States Department of Agriculture–Agricultural Research Service, Fargo, ND, United States; ^2^Department of Plant Sciences, North Dakota State University, Fargo, ND, United States; ^3^Plant Science Research Unit, United States Department of Agriculture–Agricultural Research Service, Raleigh, NC, United States; ^4^Department of Plant Pathology, North Dakota State University, Fargo, ND, United States; ^5^Wheat Health, Genetics, and Quality Research Unit, United States Department of Agriculture–Agricultural Research Service, Pullman, WA, United States; ^6^Department of Plant Pathology, Kansas State University, Manhattan, KS, United States

**Keywords:** wheat, homoeology, chromosome engineering, molecular markers, alien introgression, stripe rust, leaf rust, powdery mildew

## Abstract

*Aegilops markgrafii* (Greuter) Hammer is an important source of genes for resistance to abiotic stresses and diseases in wheat (*Triticum aestivum* L.). A series of six wheat ‘Alcedo’-*Ae. markgrafii* chromosome disomic addition lines, designated as AI(B), AII(C), AIII(D), AV(E), AIV(F), and AVIII(G) carrying the *Ae*. *markgrafii* chromosomes B, C, D, E, F, and G, respectively, were tested with SSR markers to establish homoeologous relationships to wheat and identify markers useful in chromosome engineering. The addition lines were evaluated for resistance to rust and powdery mildew diseases. The parents Alcedo and *Ae. markgrafii* accession ‘S740-69’ were tested with 1500 SSR primer pairs and 935 polymorphic markers were identified. After selecting for robust markers and confirming the polymorphisms on the addition lines, 132 markers were considered useful for engineering and establishing homoeologous relationships. Based on the marker analysis, we concluded that the chromosomes B, C, D, E, F, and G belong to wheat homoeologous groups 2, 5, 6, 7, 3, and 4, respectively. Also, we observed chromosomal rearrangements in several addition lines. When tested with 20 isolates of powdery mildew pathogen (*Blumeria graminis* f. sp. *tritici*) from five geographic regions of the United States, four addition lines [AIII(D), AV(E), AIV(F), and AVIII(G)] showed resistance to some isolates, with addition line AV(E) being resistant to 19 of 20 isolates. The addition lines were tested with two races (TDBJ and TNBJ) of the leaf rust pathogen (*Puccinia triticina*), and only addition line AI(B) exhibited resistance at a level comparable to the *Ae. markgrafii* parent. Addition lines AII(C) and AIII(D) had been previously identified as resistant to the Ug99 race group of the stem rust pathogen (*Puccinia graminis* f. sp. *tritici*). The addition lines were also tested for resistance to six United States races (PSTv-4, PSTv-14, PSTv-37, PSTv-40, PSTv-51, and PSTv-198) of the stripe rust pathogen (*Puccinia striiformis* f. sp. *tritici*); we found no resistance either in Alcedo or any of the addition lines. The homoeologous relationships of the chromosomes in the addition lines, molecular markers located on each chromosome, and disease resistance associated with each chromosome will allow for chromosome engineering of the resistance genes.

## Introduction

*Aegilops markgrafii* (Greuter) Hammer (synonym *Ae*. *caudata* L., 2*n* = 2*x* = 14, genome CC), is one of the most important diploid wild relatives of wheat (*Triticum aestivum* L., 2*n* = 6*x* = 42, AABBDD genomes) because it carries resistance to powdery mildew [caused by *Blumeria graminis* f. sp. *tritici* (DC.) Speer], leaf rust (*Puccinia triticina* Erikss.), stem rust (*Puccinia graminis* Pers.: Pers. f. sp. *tritici* Eriks. and E. Henn.) and stripe rust (*Puccinia striiformis* Westend. f. sp. *tritici* Eriks.) (Valkoun et al., 1985; Dyck et al., 1990; Schubert and Blüthner, 1995; Xu et al., 2009; Weidner et al., 2012). A set of chromosome disomic addition lines carrying *Ae. markgrafii* accession ‘S 740-69’ chromosomes B, C, D, E, F, and G in wheat variety ‘Alcedo’ were developed by Schubert and Blüthner (1992, 1995). This set of disomic addition lines can serve as an alternate and direct genetic source for wheat germplasm enhancement. An addition line for chromosome A is absent from this set, and Niu et al. (2011) found that none of the six addition lines carried high-molecular-weight glutenins from *Ae. markgrafii*, suggesting that chromosome A may be homoeologous to group 1. Friebe et al. (1992) noted results from unpublished studies which support the conclusion that chromosome A belongs to group 1. Danilova et al. (2014, 2017) determined that chromosome A of *Ae. markgrafii* is homoeologous to the group 1 chromosomes of wheat by using fluorescence *in situ* hybridization (FISH) with cDNA probes.

Xu et al. (2009) identified two Alcedo-*Ae. markgrafii* S740-69 addition lines, AII(C) and AIII(D), that conditioned resistance to the Ug99 race group of the stem rust pathogen, the most virulent races appearing in Africa. To transfer these alien genes from the addition lines to wheat in a short period of time, detailed information concerning the homoeology between wheat and the added *Ae. markgrafii* chromosomes will be very useful. There are several ways to establish the homoeologous relationships between wheat and its wild relatives, including C-banding (Friebe et al., 1992), isozyme analysis (Schmidt et al., 1993), molecular marker analysis (Peil et al., 1998), sequential fluorescence *in situ* hybridization (FISH), and genomic *in situ* hybridization (GISH) (Xu et al., 2016). In addition, marker assisted selection has become a useful tool for the gene introgression process (Niu et al., 2011). Friebe et al. (1992) and Schmidt et al. (1993) used isozymes and the C-banding technique, respectively, to determine homoeologous relationships of the six addition lines, and determined that the chromosome in lines AII(C), AIII(D), and AIV(F) belonged to group 5, 6, and 3, respectively, but homoeologous relationships of chromosomes in lines AV(E), AI(B), and AVIII(G) were not clearly established. Peil et al. (1998) tested 88 SSR markers and identified only 20 that were useful to distinguish the *Ae. markgrafii* chromosomes; and because the marker number was less than 4 for each chromosome, the results did not indicate homoeology. In addition to homoeologous relationships of each added chromosome, knowledge of the Alcedo genetic background is needed. For example, Alcedo is a major donor of stripe rust resistance (Jagger et al., 2011), and in attempting to transfer stripe rust resistance from *Ae. markgrafii*, detailed information about Alcedo is important to ensure that the stripe rust resistance is from *Ae. markgrafii* and not Alcedo.

Simple sequence repeats (SSRs) have become very useful and desirable molecular markers because they are often codominant, highly reproducible, frequent in most eukaryotes, and have high allelic diversity (Mohan et al., 1997). With the development of sequencing technology, more and more SSRs (over 3000) are available for marker analysis in wheat, and many genetic maps featuring SSR markers are available for reference. Screening for polymorphisms between the parents using additional SSRs will help to determine the homoeologous relationships and the polymorphic markers can subsequently be used for marker-assisted gene introgression. Sequential FISH and GISH will produce additional chromosome constitution information for the addition lines. Our objectives in this study were to use additional SSR markers and sequential FISH and GISH to characterize Alcedo and its six *Ae. markgrafii* addition lines, determine the homoeologous relationships of the chromosomes, and develop useful SSR markers for marker assisted selection.

## Materials and Methods

### Plant Material

Wheat cultivar ‘Alcedo,’ *Ae*. *markgrafii* (Greuter) Hammer (accession S740-69), the Alcedo-*Ae*. *markgrafii* amphiploid (W0492), and six Alcedo-*Ae*. *markgrafii* S740-69 disomic addition lines AI, AII, AIII, AV, AIV, and AVIII carrying the *Ae*. *markgrafii* chromosomes B, C, D, E, F, and G, respectively (Schmidt et al., 1993) were used for this study. A line carrying chromosome A was not available for this study. The original seed stocks of these lines were kindly provided by Dr. Richard R.-C. Wang, USDA-ARS Forage and Range Research Laboratory, Logan, UT, United States.

### Fluorescence *in situ* Hybridization

Root-tips of plants were prepared for FISH following the procedure described by Xu et al. (2016). This included pretreatment of root tips in ice water for 20–24 h, fixation in ethanol-acetic acid (3:1 ratio), pretreatment in 1% acetocarmine, and squashing on a slide using 45% acetic acid. Slides were examined to select samples with good preparations, and cover glasses were removed. Prepared slides were incubated in 100 μg/mL RNase in 2× saline sodium citrate (SSC) at 37°C for 1 h, then denatured in 70% formamide in 2× SSC at 72°C for 2 min followed by dehydration in a chilled graded ethanol series (70%, 95%, and 100%) at -20°C each for 5 min.

Multi-color FISH was carried out with two probes, pAS1 carrying about 1 kb of repeat sequence from *Ae. tauschii* Cosson (Rayburn and Gill, 1986) and labeled with digoxigenin-11-dUTP (Roche Diagnostics, Mannheim, Germany), and pSC119.2 carrying a highly repeated sequence from rye (*Secale cereale* L.) and labeled with biotin-16-dUTP (Enzo Life Sciences, Inc., Farmingdale, NY, United States). The two probes were equally mixed before hybridization and then added to the hybridization mix (15 μL formamide, 6 μL dextran sulfate, 3 μL 20× SSC, and 3 μL single stranded DNA). Fifteen microliters of hybridization mix were added to each slide and slides were covered with cover slips for incubation overnight. The slides were then washed as described by Xu et al. (2016). The fluorescent signals were detected with anti-digoxigenin-rhodamine (Roche Diagnostics) and fluorescein isothiocyanate-conjugated avidin (FITC-avidin) (Vector Laboratories, Inc., Burlingame, CA, United States) for both probes. The slides were mounted with VECTORSHIELD Antifade Mounting Medium (Vector Laboratories) containing 4′,6-diamidino-2-phenylindole (DAPI) (Sigma, St. Louis, MO, United States). The slides were examined under a Zeiss Axioplan 2 Imaging Research Microscope (Carl Zeiss Light Microscopy, Germany). The GISH images were captured using an Axiocam HRm CCD (charge-coupled device) camera (Carl Zeiss Light Microscopy) and analyzed using imaging software AxioVision Release 4.5 (Carl Zeiss Light Microscopy).

### Genomic *in situ* Hybridization

After FISH, the slides were washed in 0.1× SSC with 0.5% formamide three times each for 10 min at 42°C, then 2× SSC twice each for 10 min, then in 4× SSC overnight at room temperature. The slides were sequentially dehydrated in 70%, 95%, and 100% ethanol each for 5 min. Total genomic DNA from *Ae. markgrafii* was used as probe and labeled with biotin-16-dUTP by nick translation (Enzo Life Sciences, Inc.). Sheared genomic DNA from Chinese Spring was used for blocking. Detailed procedures of the chromosome preparation and hybridization were previously described by Xu et al. (2016). GISH signals were detected with FITC-avidin (Vector Laboratories). The slides were mounted with VECTORSHIELD Antifade Mounting Medium (Vector Laboratories) containing propidium iodide (PI) (Vector Laboratories) and were observed under the Zeiss Axioplan 2 Imaging Research Microscope for GISH analysis as described above. Photographs were captured using the Axiocam HRm CCD camera and analyzed using the imaging software AxioVision Release 4.5 for GISH analysis as described above.

### Karyotype Analysis of *Ae. markgrafii* Chromosomes

The chromosome spreads from GISH and FISH analyses were used for karyotypic analysis of each of the *Ae. markgrafii* chromosomes in the six addition lines. Each of the *Ae. markgrafii* chromosomes was measured for lengths of short and long arms from at least 20 cells using the “Measure Length” tool in the imaging software AxioVision Release 4.5 (Carl Zeiss Light Microscopy). Total length of each *Ae. markgrafii* chromosome was calculated by adding the averages of long and short arms. The arm ratio (long arm/short arm) of each *Ae. markgrafii* chromosome was calculated from the lengths of short and long arms.

### SSR Marker Analysis

DNA extraction from fresh leaves and SSR marker genotyping were carried out according to the procedure outlined by Niu et al. (2011). Markers studied included SSRs from the BARC (Song et al., 2005), GWM (Röder et al., 1998), WMC (Somers et al., 2004), CFA (Sourdille et al., 2003), GDM (Pestsova et al., 2000), CFD (Guyomarc’h et al., 2002), DuPw (Eujayl et al., 2002), KSM (Yu et al., 2004), CNL (Yu et al., 2004), and AC (Barkley et al., 2006) groups. Markers were assigned to chromosomes and chromosome groups based on locations reported in the citations or based on a search for the markers in the GrainGenes database^1^. DNA fragments were amplified by polymerase chain reaction (PCR) at an annealing temperature of 50°C and labeled with four different fluorescent dyes (6-FAM, VIC, NED, and PET). Amplified PCR products were separated by capillary electrophoresis using the ABI 3130xl Genetic Analyzer (Applied Biosystems, Forster City, CA, United States) according to the procedures of Chao et al. (2007). The genotype calls were analyzed using GeneMapper software v3.7 (Applied Biosystems).

### Evaluation of *Ae. markgrafii* Addition Lines for Resistance to Leaf Rust, Stripe Rust, and Powdery Mildew

Wheat cultivar Alcedo, *Ae*. *markgrafii* accession S740-69, Alcedo-*Ae*. *markgrafii* amphiploid W0492, six disomic addition lines, and Chinese Spring were included in tests for resistance to leaf rust, stripe rust, and powdery mildew.

Leaf rust resistance was evaluated at North Dakota State University (Fargo, ND, United States) followed the procedures of Kertho et al. (2015). Two *P. triticina* races, TDBJ+*Lr21&Lr28* and TNBJ, were used to evaluate the genotypes. Race TDBJ+*Lr21&Lr28* produces a high infection type on *Lr21* and *Lr28*, while TNBJ has a high infection type on *Lr9.* The experiment was conducted using a randomized complete block design with two replicates, with the entire experiment being repeated for each race as described by Kertho et al. (2015). Approximately five seeds per genotype were planted in a greenhouse set at 22°C/18°C (day/night) with 16-h photoperiod. Ten-day-old seedlings were inoculated by spraying fresh urediniospores suspended in a light mineral oil (Soltrol-170, Phillips Petroleum, Bartlesville, OK, United States). Following inoculation, plants were placed into a darkened dew chamber maintained at 20°C for 16–24 h. Following the incubation period, the plants were removed to a greenhouse maintained at 20°C with a normal 16/8 h day/night photoperiod. Genotypes were scored for infection types (ITs) at 12–14 days post inoculation using the 0–4 scale (McIntosh et al., 1995). Infection types of 2 or lower were considered resistant, and ITs 3 or higher were considered susceptible.

Resistance to stripe rust was evaluated at USDA-ARS, Wheat Health, Genetics, and Quality Research Unit, Pullman, WA, United States, using six *P. striiformis* f. sp. *tritici* races, PSTv-4, PSTv-14, PSTv-37, PSTv-40, PSTv-51, and PSTV-198 (Wan and Chen, 2014; Wan et al., 2016). For each race test, 5–10 seeds per line were planted and seedlings at the two-leaf stage were uniformly inoculated with a mixture of urediniospores with talc at a 1:20 ratio and kept in a dew chamber for 24 h at 10°C and 100% relative humidity without light. The inoculated plants were them moved to a growth chamber at a diurnal temperature cycle gradually changing from 4°C at 2:00 am to 20°C at 2:00 pm and a diurnal cycle of 8 h dark/16 light corresponding to the low/high temperature cycle (Wan and Chen, 2014). Plants were scored 20 days post inoculation using the 0–9 scale of Line and Qayoum (1992).

Powdery mildew tests were conducted at the USDA-ARS, Plant Science Research Unit, Raleigh, NC, using the detached-leaf method as described by Worthington et al. (2014). Twenty isolates representing differing United States regional virulence profiles were used for tests. These isolates originated from nine US states of the Southeast, Mid-Atlantic, Great Lakes, and Great Plains wheat growing regions. To simplify presentation, two Montana isolates are included as “Great Plains” isolates despite originating west of the Great Plains because they exhibit a similar virulence profile to isolates from the Great Plains (Cowger et al., 2018). Inoculations were performed following Worthington et al. (2014). In brief, two 1.5-cm detached leaf segments from each genotype were floated on 0.5% water agar containing 50 mg L^-1^ benzimidazole in a Petri plate. Each plate also contained four replicate leaf segments of a susceptible wheat cultivar as a positive control. Four replicate Petri plates per host genotype were inoculated with each isolate of *B. graminis* f. sp. *tritici*. The plates were then placed in a growth chamber set to 18°C with an 11-h photoperiod. Disease reactions were scored 10 days post-inoculation using the 0–9 scale of Leath and Heun (1990). Reactions were then classified as resistant (R), intermediate (I), or susceptible (S) based on whether the predominant reaction among the replicate plates was <4, 4 to 6, or >6, respectively.

## Results

### GISH and FISH Analysis of Six Alcedo-*Ae*. *markgrafii* Disomic Addition Lines

**FIGURE 1:**
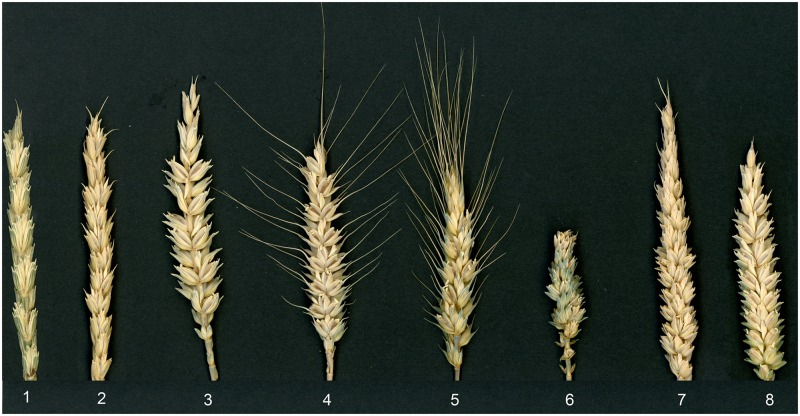
The morphology of the spikes of Alcedo, the amphiploid between Alcedo and *Aegilops markgrafii*, and six wheat addition lines carrying the alien chromosomes from *Ae. markgrafii.* 1, amphiploid, 2, AI(B); 3, AII(C); 4, AIII(D); 5, AV(E); 6, AIV(F); 7, AVIII(G); 8, Alcedo.

The six Alcedo-*Ae*. *markgrafii* disomic addition lines, AI(B) through AVIII(G), were examined for differences in spike morphologies (Figure 1). We observed that AI(B) is unique in its non-free threshing spikes, AII(C) has large glumes, AIII(D) and AV(E) have awns, and AIV(F) has club spikes with brittle rachis. Lines AV(E) and AVIII(G) have sterile spikelets on the top and in the upper half portion of the spikes, respectively (Table 1).

**Table 1:** Karyotypic characteristics of *Aegilops markgrafii* chromosomes and the spike agronomic traits of the six Alcedo-*Ae*. *markgrafii* S740-69 disomic addition lines.

Addition lines	Length (μm) of *Ae*. *markgrafii* chromosome			Arm ratio (L:S)	Spike traits
			
	Long arm (L)	Short arm (S)	Total		
AI(B)	6.73	2.05	8.78	3.29	Non-free threshing
AII(C)	6.63	3.58	10.21	1.85	Large glumes
AIII(D)	5.94	2.57	8.51	2.31	Awned
AV(E)	6.32	1.67	7.99	3.79	Top of spike is sterile, awned
AIV(F)	6.36	1.50	7.86	4.24	Club spikes, brittle rachis
AVIII(G)	6.08	1.31	7.39	4.63	Top half of spike is sterile


*Non-free threshing QTLs were mapped to group 2 (Sood et al., 2009), brittle rachis QTLs located on group 3 chromosome (Nalam et al., 2006; Watanabe et al., 2006).*

**FIGURE 2:**
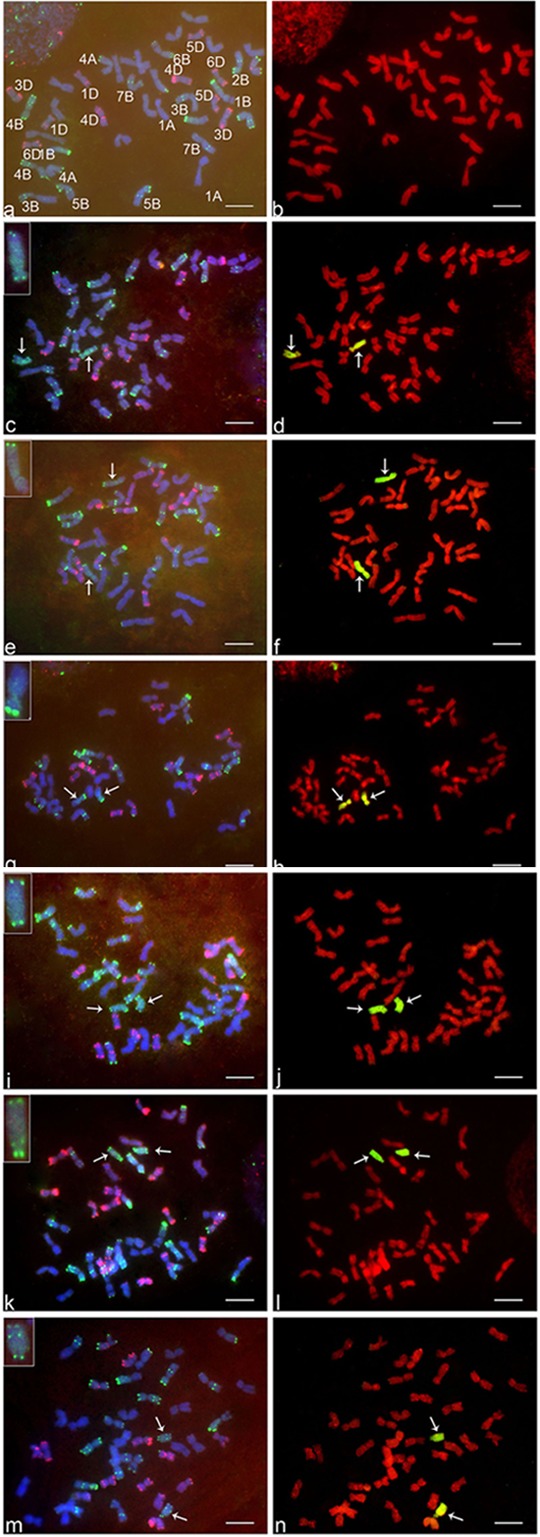
FISH and GISH on the somatic metaphase chromosomes of six addition lines and their wheat parent ‘Alcedo.’ The left side of figure **(a,c,e,g,i,k,m)** are FISH results, where red indicates pAs1 hybridization sites detected by rhodamine fluorescence and green indicates pSc119.2 hybridization sites detected by FITC fluorescence. The right sides of figure **(b,d,f,h,j,l,n)** are GISH results, where green indicates *Aegilops markgrafii* chromatin detected by FITC fluorescence. **a** and **b**, Alcedo; **c** and **d**, AI(B); **e** and **f**, AII(C); **g** and **h**, AIII(D); **i** and **j**, AV(E); **k** and **l**, AIV(F); **m** and **n**, AVIII(G). Arrows indicate the alien chromosomes. Bar represents 10 μm.

The GISH analysis (Figure 2) showed that all six lines had a mitotic chromosome number of 2*n* = 44, and in each case, only one pair of chromosomes showed a distinct green coloration (arrows) compared to the red coloration of the remaining 42 chromosomes. No structural abnormalities were observed on any of the chromosomes. These results indicated that each addition line carried only one intact chromosome pair from *Ae. markgrafii.* Karyotypic characteristics of six *Ae. markgrafii* chromosomes are listed in Table 1. The long arm to short arm ratios of the *Ae. markgrafii* chromosomes B, C, D, E, F, and G were 3.29, 1.85, 2.31, 3.79, 4.24, and 4.63, respectively (Table 1). The FISH results showed that the *Ae. markgrafii* chromosomes (arrows) in all the additions had the pSC119.2 hybridization signals in the telomeric regions in either one or both arms (Figure 2). The general morphologies of the six *Ae. markgrafii* chromosomes from GISH/FISH analysis are consistent with those from the N- and C-banded karyotypes reported by Schubert et al. (1987) and Friebe et al. (1992), respectively. By comparing to the reference karyotype developed based on *Ae. markgrafii* accession MvGB428 (Molnár et al., 2015), we found that only chromosomes C and F had the identical pSC119.2 band patterns as chromosomes 5C and 3C, respectively.

### Identification of SSR Markers Associated to *Ae. markgrafii* Chromosomes

In this study, 1,500 SSR primer pairs were used to detect polymorphism between the parents, Alcedo and *Ae. markgrafii* accession S740-69. Nine hundred and thirty-five pairs of SSRs (62.3%) amplified polymorphic bands. From those polymorphic primer pairs, SSRs located on group 1 chromosomes, the majority of the SSRs that produced dominant bands and SSRs that produced weak bands were eliminated from further analyses. As a result, only 234 robust SSRs were selected for analysis of the Alcedo*-Ae. markgrafii* addition lines. These SSRs were comprised of 27 BARCs, 58 GWMs, 72 WMCs, 14 CFAs, 16 GDMs, 34 CFDs, 5 DuPws, 4 KSMs, 3 CNL, and 1 AC. These SSRs belonged to six homoeologous groups, 52 to group 2, 45 to group 3, 35 to group 4, 47 to group 5, 24 group to 6, and 31 to group 7. Analysis of the *Ae. markgrafii* addition lines resulted in the elimination of additional SSRs. As a result, only 132 SSRs were polymorphic between the addition lines and Alcedo (Supplementary Table 1). However, many of these SSRs mapped to multiple groups (Supplementary Table 1), and therefore, in the summarized distribution of the SSRs to chromosome groups, it appears that there are more than 132 polymorphic SSRs (Table 2).

**Table 2:** The distribution of the polymorphic SSR marker belonging to different homoeologous groups in six Alcedo-*Aegilops markgrafii* disomic addition lines.

Addition lines	Number of SSR markers belonging to homoeologous group						Total
		
	2	3	4	5	6	7	
AI(B)	17	3	5	3	6	4	28
AII(C)	7	7	3	29	2	3	36
AIII(D)	4	6	3	6	12	8	29
AV(E)	4	7	3	0	3	8	19
AIV(F)	3	14	0	3	2	2	20
AVIII(G)	7	11	5	1	1	2	25
Total	42	48	19	42	26	27	132


*The total number of polymorphic markers was less than the sum of markers for the six groups because several markers mapped to more than one homoeologous group.*

The assignment of *Ae. markgrafii* chromosomes to homoeologous groups was determined based on the distribution of the polymorphic SSR markers among the addition lines. Of the 28 polymorphic markers identified for addition line AI(B) (Table 2), 17 (61%) mapped to group 2 chromosomes, suggesting that the alien chromosome in the AI(B) addition line belongs to group 2. Similar comparisons for the other five addition lines clearly indicate that the *Ae. markgrafii* chromosomes in the lines AII(C), AIII(D), and AV(F) belongs to groups 5, 6, and 3, respectively. The chromosome in line AV(E) might belong to group 7 or group 3, and the G addition chromosome might belong to group 2, 3, or 4 (Table 3).

**Table 3:** Assignment of homoeologous groups of six *Aegilops markgrafii* chromosomes derived from six Alcedo-*Ae. markgrafii* disomic addition lines.

Addition line	Homoeologous groups assigned in this study			Homoeologous groups assigned by		
			
	SSR markers	Karyotype and pSC119.2	Spike traits	Schmidt et al. (1993)	Gong et al. (2017)	Danilova et al. (2017)
AI(B)	2	-	2	4/5	1/2/3/5	2/4
AII(C)	5	5	-	5	2/5	5
AIII(D)	6	-	-	6	2/5/6	6/7
AV(E)	7/3	-	-	-	1/2/7	7
AIV(F)	3	3	2/3	3	2/3	3
AVIII(G)	3/2/4	-	-	4/3	1/2/3/4	4/2/3


*Assignment of homoeologous groups using karyotype and pSC119.2 probe was based on the *Ae. markgrafii* reference karyotype (Molnár et al., 2015).*

### Reactions of Alcedo-*Ae*. *markgrafii* Addition Lines to Leaf Rust, Stripe Rust, and Powdery Mildew

The addition lines were tested with two races (TDBJ and TNBJ) of the leaf rust pathogen (Table 4). As expected, Chinese Spring was susceptible to both races. Alcedo had an intermediate (2^+^3) or resistant (2) IT to TDBJ and TNBJ, respectively; indicating that it carries at least one leaf rust resistance gene. The *Ae*. *markgrafii* parent (S740-69) was highly resistant to both races, with an immune response. For the six disomic addition lines, only AI(B) exhibited resistance, with a level of resistance similar to S740-69. The B chromosome appears to be a good source of leaf rust resistance.

**Table 4:** Infection types (IT) observed on *Aegilops markgrafii* addition lines when tested with two races of the leaf rust pathogen (*Puccinia tritcina, Pr*) and one race of stripe rust pathogen (*Puccinia striiformis* f. sp. *tritici, Pst*).

Line or genotype	IT to *Pr* race		IT to *Pst* race
		
	TDBJ	TNBJ	PSTv-14
Chinese Spring	32	32	8
Alcedo	2^+^3	2	8
S740-69 (*Ae. markgrafii*)	0	0	0
W0492 (amphidiploid)	-	-	8
AI(B)	0;	;	8
AII(C)	3	3	8
AIII(D)	2	3	8
AV(E)	3	3	8
AIV(G)	3	3	8
AVIII(G)	3	3^-^	8


*The value reported is the higher of the two reps.*

*Five other stripe rust races tested on the lines produced identical results.*

The addition lines and parents were tested with six races of the stripe rust pathogen (Table 4). Although Alcedo had been reported to carry two genes for stripe rust resistance, Alcedo was observed to be highly susceptible (IT 8) to all six US races. For the remaining parents and addition lines, the *Ae. markgrafii* parent (S740-69) was immune, but all other lines were highly susceptible. The amphidiploid W0492 was included in the stripe rust tests, and it expressed an IT similar to Alcedo and all addition lines; and this indicated that the resistance in S740-69 could not be confirmed to any of the seven *Ae. markgrafii* chromosomes.

When tested with powdery mildew (Table 5), Chinese Spring was susceptible to all 20 isolates, while Alcedo was susceptible to 19 isolates and had an intermediate reaction to isolate *MTG1-1a*. In contrast, S740-69 was resistant to all 20 isolates, indicating *Ae. markgrafii* was an excellent source of powdery mildew resistance. Among the addition lines, AI(B) and AII(C) were susceptible to almost all isolates, indicating that resistance was not contributed by the B and C chromosomes. The remaining four addition lines had varying levels of resistance. Line AV(E) had resistance to 19 of 20 isolates and an intermediate reaction to isolate *MSG-D-1-5*. Line AIII(D) was resistant to all eight Great Plains isolates, but had a mixture of R, I, and S reactions to isolates from the other geographical regions. Line AIV(F) also had resistance to all Great Plains isolates, but AIV(F) had a different mixture of R, I, and S reactions to other isolates as compared to AIII(D). AVIII(G) had a mixture of reactions without regard to the region of origin and had more intermediate reactions than the other addition lines. In summary, the E chromosome conditioned resistance to nearly all isolates, the D and F chromosomes conditioned resistance to the Great Plains isolates and some isolates from other regions, and the G chromosome conditioned resistance to some isolates without regard to the region of origin.

**Table 5:** Reactions of six Alcedo-*Aegilops markgrafii* addition lines, their parents, and the Chinese Spring check when tested with 20 isolates of powdery mildew pathogen collected from nine states and four regions of the United States.

Isolate	State	Region	Line or genotype								
			
			Chinese Spring	Alcedo	S740-69	AI(B)	AII(C)	AIII(D)	AV(E)	AIV(F)	AVIII(G)
*GAP-A-2-3*	GA	Southeast	S	S	R	S	S	S/I	R	I	R
*GAP-B-2-2*	GA	Southeast	S	S	R	S/I	S	S	R	S	R
*MSG-A-3-1*	MS	Southeast	S	S	R	S	S	R	R	R	I
*MSG-D-1-5*	MS	Southeast	S	S	R	S	S	S	I	S	R/I
*NCF-D-1-1*	NC	Mid-Atlantic	S	S	R	S	S	R	R	I	I
*NCC-B-1-3*	NC	Mid-Atlantic	S	S	R	S	S	I	R	S	R
*NYA-E-3-3*	NY	Great Lakes	S	S	R	S	S	-	R	S	R
*NYB-E-1-2*	NY	Great Lakes	S	S	R	S	S	S	R	S	S
*PAF(14)-D-1-2*	PA	Great Lakes	S	S	R	S	S	S	R	S	R
*PAF-E-2-2*	PA	Great Lakes	S	S	R	S	S	R	R	R	I
*MIR(14)-D-3-3*	MI	Great Lakes	S	S	R	S	S	R	R	I	R
*MIR(14)-E-1-3*	MI	Great Lakes	S	S	R	S	S	R	R	I	R
*MTG1-3a*	MT	Great Plains	S	S	R	S	S/I	R	R	R	S/I
*MTG1-1a*	MT	Great Plains	S	I	R	S	S	R	R	R	R
*OKH-A-2-3*	OK	Great Plains	S	S	R	S	S	R	R	R	R/I
*OKS-A-2-2*	OK	Great Plains	S	S	R	S	S	R	R	R	I
*OKS-B-2-2*	OK	Great Plains	S	S	R	S	S	R	R	R	I
*NEI3-1*	NE	Great Plains	S	S	R	S	S	R	R	R	I
*NEI1-3*	NE	Great Plains	S	S	R	S	S	R	R	R	I
*NEI5-5*	NE	Great Plains	S	S	R	S	S/I	R	R	R	I


*Average scores were less than 4, between 4 and 6, and higher than 6 for resistant (R), intermediate (I), and susceptible (S), respectively.*

## Discussion

In assigning SSRs to specific *Ae. markgrafii* chromosomes, the addition lines must exhibit a high level of homogeneity relative to Alcedo to exclude detection of polymorphisms on the wheat chromosomes. Friebe et al. (1992) concluded from C-banding results that the Alcedo-*Ae. markgrafii* addition lines were not in a pure Alcedo background. This is supported by the results of Niu et al. (2011), who studied HMW glutenin subunits in the six addition lines. They found addition line AIII(D) differed from Alcedo at all three *Glu-1* loci, which indicated an additional wheat genotype in the parentage of AIII(D) rather than the presence of biotypes in Alcedo. Variability in the wheat background of the addition lines complicates determination of the origin of the observed polymorphisms. For example, in AIII(D) the evidence suggests that the *Ae. markgrafii* chromosome is homoeologous to group 6, but additional markers also mapped to all the other chromosome groups.

The observed variability in the assignment of molecular markers to chromosomes indicates the presence of chromosomal rearrangements. Studies by Danilova et al. (2017) and Gong et al. (2017) found that the Alcedo-*Ae. markgrafii* additions lines carried several inversions and translocations. While both studies found a high level of rearrangement, the two studies did not agree on the rearrangements carried by each chromosome (Table 3). For example, Danilova et al. (2017) concluded that chromosome D was mainly a group 6 chromosome with the long arm telomere composed of a translocated 7CL telomeric region. In contrast, Gong et al. (2017) concluded that the rearrangements in chromosome D involved chromosomes 2C, 5C, and 6C.

For each addition line, we observed markers that were not associated with the homoeologous group identified for that line. For example, of the 28 polymorphic markers associated with addition line AI(B), 17 were group 2 markers, and 11 markers were therefore not associated with group 2. There is more than one possible explanation for the markers that do not fit with the alien chromosome. Some of these markers may represent polymorphisms present in the addition lines that were not eliminated during backcrossing to Alcedo, and therefore these markers would not be associated with the alien chromosome. Some may actually be associated with the alien chromosome but have simply not been previously identified to that homoeologous group. Finally, some may be associated with the alien chromosome, but the rearrangements present results in markers being identified with multiple homoeologous groups. For example, Danilova et al. (2017) concluded that the *Ae. markgrafii* chromosome D carried a group 6/7 rearrangement, and 19 of the 29 markers we observed for AIII(D) would fit with this rearrangement. Similarly, Danilova et al. (2017) concluded that *Ae. markgrafii* chromosome G carried a 4/2/3 rearrangement, and 21 of the 25 markers we observed fit this rearrangement. Therefore, our results seem to fit well with the conclusions of Danilova et al. (2017). However, considering the high levels of rearrangements in the *Ae. markgrafii* genome, it is possible that the differences in the present study from Danilova et al. (2017) and Gong et al. (2017) may represent observational differences, with additional rearrangements yet to be discovered. The GISH and FISH analysis showed that the *Ae. markgrafii* chromosomes in AII(C) and AIV(F) (Figure 2) are morphologically most like chromosomes 5C and 3C of the reference karyotype (Molnár et al., 2015), respectively. Taken together, the spike traits (Table 1), molecular marker data (Table 2), and FISH and GISH analyses (Figure 2) indicated that *Ae. markgrafii* chromosomes in AI(B), AII(C), AIII(D), AV(E), AIV(F), and AVIII(G) belong to groups 2, 5, 6, 7, 3, and 4, respectively.

Spike traits were recorded for each *Ae. markgrafii* addition line, and in two instances, the observed trait (Table 1) corresponded with the molecular marker data. In AI(B), spikes were non-free threshing. Genes for tenacious glume (*Tg*) have been identified on group 2 chromosomes (Simonetti et al., 1999; Jantasuriyarat et al., 2004; Sood et al., 2009; Faris et al., 2014; Katkout et al., 2014). Genes for brittle rachis have been located to group 3 chromosomes (Nalam et al., 2006; Watanabe et al., 2006). These observations agree with the conclusion that *Ae. markgrafii* chromosomes B and F are homoeologous to group 2 and 3, respectively. Other spike traits did not yield useful homoeology information. For example, large glumes and club spikes were associated with chromosomes C (group 5) and F (group 3), respectively. The large glume trait of *T. polonicum* has been mapped to group 7 chromosomes (Watanabe, 1999), while the club spike trait is a group 2 characteristic (Johnson et al., 2008). The failure to observe corresponding results between the molecular data and the morphological traits may represent either incomplete knowledge of the trait, impurity of the Alcedo background, or may indicate that the chromosomes in question carry chromosomal rearrangements.

We observed resistance to leaf rust conditioned by chromosome B of *Ae. markgrafii*. In contrast, Gong et al. (2017) found that chromosome D conditioned resistance to leaf rust, while chromosome B provided no leaf rust resistance. It is possible that the differences in these two studies may represent differential response to races. However, Iqbal et al. (2007) transferred leaf rust resistance from *Ae. markgrafii* to wheat chromosome arm 2AS, and they noted that chromosome B was the likely source of this gene.

Alcedo has been reported to carry two major and two minor genes conferring adult-plant resistance to stripe rust (Jagger et al., 2011). When we tested the addition lines for resistance to six US races of *P. striiformis* f. sp. *tritici* in the seedling stage, we observed a susceptible reaction on Alcedo, all addition lines, and the amphidiploid. Our results were consistent with Jagger et al. (2011) that Alcedo was susceptible in the seedling stages to the United Kingdom isolates used in the field tests. However, the seedling tests could not detect the adult-plant resistance in Alcedo. Nevertheless, the seedling data showed that the addition lines did not get any genes from *Ae. markgrafii* for all-stage resistance against the current predominant and most virulent races in the United States. Further tests of the lines with the races at the adult-plant stage or in the field are needed to determine if the addition lines inherited any adult-plant resistance genes from Alcedo and/or from *Ae. markgrafii*.

For resistance to powdery mildew, Gong et al. (2017) tested the six addition lines and parents using mixed races of the pathogen in China. They identified only line AV(E) as carrying resistance from *Ae. markgrafii*. However, we found that four addition lines, AIII(D), AV(E), AIV(F), and AVIII(G), carried powdery mildew resistance and *Ae. markgrafii* accession S740-69 was resistant to all 20 isolates tested in our study. The resistance conferred by chromosomes D, F, and G was generally confined to isolates originating from a geographical region and thus restricts their adaptability. The E chromosome conferred resistance to 19 of the 20 powdery mildew isolates in the test, making it particularly attractive for alien gene introgression.

We report here tests for powdery mildew, stripe rust, and leaf rust resistance. The previous study of Xu et al. (2009) identified chromosomes C and D as carrying resistance to the Ug99 race group of the stem rust pathogen. Therefore, each of the six addition lines carries resistance to at least one fungal disease, making this a rich resource for gene introgression. Alien gene introgression is very valuable for introduction of new traits into wheat. Historically these introgressions were the product of homoeologous recombination or radiation induced chromosomal breakage which usually required standard cytogenetic techniques. With the incorporation of molecular markers as a tool to select recombinants, induced homoeologous recombination is much more effective than techniques that relied on cytogenetic observation. This study therefore identifies not only which lines carry disease resistance genes, but also identifies markers that can be used to detect recombination. By using the SSR markers associated with *Ae. markgrafii* chromosome D, we recently introgressed a new gene for Ug99 resistance from AIII(D) into common wheat (Xu et al., 2017).

## Author Contributions

SX conceived and planned this study. ZN, DK, and SC conducted marker analysis. ZN, XWC, BF, BG, and SX conducted molecular and cytogenetic analysis on alien chromosomes. RW and CC performed assay for powdery mildew resistance. MB and JR conducted leaf rust test. XMC conducted stripe rust test. ZN, DK, and SX wrote the manuscript. All authors reviewed and edited the manuscript.

## Conflict of Interest Statement

The authors declare that the research was conducted in the absence of any commercial or financial relationships that could be construed as a potential conflict of interest.

## Footnotes

1 http://wheat.pw.usda.gov/cgi-bin/GG3/browse.cgi?class=marker
