# Supplementary material for: Molecular and Cytogenetic Characterization of Six Wheat-Aegilops markgrafii Disomic Addition Lines and Their Resistance to Rusts and Powdery Mildew
Source: Front Plant Sci. 2018 Nov 8;9:1616. doi: 10.3389/fpls.2018.01616 (PMC6236143; doi:10.3389/fpls.2018.01616)
Supplement: Supplementary file 1 [file Table_1.DOCX]

| **Supplemental Table 1 ǀ** List of 132 simple sequence repeat (SSR) markers that were polymorphic between Alcedo and each *Aegilops markgrafii* disomic addition line, and their chromosome locations in wheat and *Ae. markgrafii*. | | | | |
| --- | --- | --- | --- | --- |
| SSR marker | Chromosome location | | *Ae. markgrafii* chromosomes | |
| cfa2201 |  | 2A/2B |  | B |
| wmc63 |  | 2A |  | B |
| barc349 |  | 2B |  | B |
| barc5 |  | 2A/6D/7D |  | B |
| barc95 |  | 2D/7B |  | B |
| cfa2278 |  | 2B |  | B |
| cfd116 |  | 2D |  | B |
| cfd71 |  | 4A/4D |  | B |
| cfd77 |  | 2D |  | B |
| gwm455 |  | 2D/6B |  | B |
| ksm019 |  | 7A |  | B |
| wmc154 |  | 2B |  | B |
| wmc245 |  | 2B/2D |  | B |
| wmc344 |  | 2B |  | B |
| wmc601 |  | 2D/2A |  | B |
| wmc661 |  | 2B |  | B |
| gwm165 |  | 4A/4B/4D |  | B |
| gwm319 |  | 2B/7A |  | B |
| gwm494 |  | 4A/6A/1B/3A/5D |  | B |
| gwm512 |  | 2A |  | B |
| gwm192 |  | 4A/4B/4D/5D |  | B |
| gwm219 |  | 6B |  | B |
| wmc524 |  | 5A |  | B/C/D/F |
| cfd42 |  | 6D |  | B/C/D/G |
| cfd53 |  | 2D |  | B/D |
| gdm125 |  | 4D/1B/6A |  | B/D |
| gdm38 |  | 3D/1B |  | B/D/F |
| gwm566 |  | 3B |  | B/E/G |
| barc117 |  | 5AS |  | C |
| barc140 |  | 5BL/2B/5D |  | C |
| barc59 |  | 5BL/2D |  | C |
| cfa2070 |  | 5B |  | C |
| cfa2104 |  | 5A/5B/5D |  | C |
| cfd156 |  | 5B/5D |  | C |
| cfd18 |  | 5D |  | C |
| cfd189 |  | 5D |  | C |
| cfd266 |  | 5D |  | C |
| cfd57 |  | 5D |  | C |
| cfd7 |  | 5B/5D |  | C |
| gdm116 |  | 5B/5D |  | C |
| gdm43 |  | 5D/3A |  | C |
| gdm68 |  | 5A/5B/5D |  | C |
| gdm88 |  | 4A/7D |  | C |
| gdm99 |  | 5D/3D/7D |  | C |
| gwm190 |  | 5D |  | C |
| gwm212 |  | 5D |  | C |
| gwm272 |  | 5D |  | C |
| gwm654 |  | 5D |  | C |
| wmc149 |  | 5B/2A/2B/2D |  | C |
| wmc215 |  | 5A/5D/3A |  | C |
| wmc261 |  | 2A/2B/1D/3B/7B |  | C |
| wmc327 |  | 5A |  | C |
| wmc468 |  | 4A |  | C |
| wmc75 |  | 5B |  | C |
| gwm159 |  | 5B/5D |  | C |
| gwm205 |  | 5A/5D |  | C |
| gwm565 |  | 4A/5D |  | C |
| gwm639 |  | 5A/5B/5D |  | C |
| barc101 |  | 2BL/3B/5A/6B |  | C |
| gwm52 |  | 2A/2B/2D/3D |  | C/D/G |
| cfa2191 |  | 3B |  | C/E |
| gdm29 |  | 2D |  | C/G |
| ac1 |  | 7B |  | D |
| cfd190 |  | 6A/6B/6D |  | D |
| cfd30 |  | 6A/1A/4A/7D |  | D |
| cfd37 |  | 6D/5D |  | D |
| cfd47 |  | 6D/6B/5A |  | D |
| cfd67 |  | 5D |  | D |
| cfd76 |  | 6D |  | D |
| dupw217 |  | 6B |  | D |
| dupw398 |  | 7B |  | D |
| gdm147 |  | 6B |  | D |
| ksm061 |  | 6A |  | D |
| wmc139 |  | 7A |  | D |
| wmc232 |  | 4A/7B |  | D |
| wmc552 |  | 3D |  | D |
| wmc658 |  | 2A |  | D |
| wmc773 |  | 5B/6D |  | D |
| gwm332 |  | 7A |  | D |
| wmc505 |  | 3A/3B/3D |  | D |
| wmc621 |  | 6A/6B |  | D/E |
| wmc607 |  | 7A |  | D/E/F |
| cfd6 |  | 2A/3B/7A |  | D/E/F |
| gwm314 |  | 3D |  | D/F |
| barc4 |  | 5BL/5BS |  | D/F |
| barc231 |  | 2AS/7BS |  | E |
| barc324 |  | 3A |  | E |
| barc42 |  | 3D |  | E |
| barc60 |  | 1B/4B |  | E |
| cfa2049 |  | 7A |  | E |
| cfa2234 |  | 3A |  | E |
| wmc335 |  | 7B/6A |  | E |
| wmc457 |  | 4D |  | E |
| wmc473 |  | 4D/6B/7D |  | E |
| wmc488 |  | 7A/7D |  | E |
| gwm573 |  | 7A/7B |  | E |
| gwm601 |  | 4A |  | E |
| gwm294 |  | 2A |  | E/F |
| barc147 |  | 3B/3D/2B |  | E/G |
| barc284 |  | 3A |  | F |
| barc294 |  | 3A |  | F |
| dupw227 |  | 3A |  | F |
| gdm128 |  | 3D |  | F |
| wmc231 |  | 3B |  | F |
| wmc532 |  | 3A |  | F |
| gwm2 |  | 3A/3D |  | F |
| gwm674 |  | 3A |  | F |
| cfa2134 |  | 3A/3B |  | F |
| cfd152 |  | 3D |  | F |
| cfd79 |  | 3A/3B/3D |  | F |
| cfd188 |  | 6D |  | F |
| barc142 |  | 2D/5A/5B/6A |  | F |
| barc314 |  | 3A |  | G |
| cfd168 |  | 2A/2D |  | G |
| cfd54 |  | 4B/4D |  | G |
| cnl126 |  | 3B |  | G |
| dupw207 |  | 2B |  | G |
| gdm129 |  | 4D |  | G |
| gdm150 |  | 7D |  | G |
| gwm292 |  | 5D |  | G |
| wmc153 |  | 1D/3A |  | G |
| wmc167 |  | 2D/2B |  | G |
| wmc322 |  | 3A/3B/7B |  | G |
| wmc41 |  | 2D |  | G |
| wmc418 |  | 3B/3D |  | G |
| wmc48 |  | 4A/4B/4D |  | G |
| wmc559 |  | 3A |  | G |
| wmc617 |  | 4A/4B/4D |  | G |
| gwm113 |  | 4B |  | G |
| gwm260 |  | 7A |  | G |
| gwm32 |  | 3A |  | G |
| gwm3 |  | 3D |  | G |
| ^a^Chromosome locations in wheat are as listed in the GrainGenes database (<https://wheat.pw.usda.gov/cgi-bin/GG3/browse.cgi?class=marker>, accessed on August 15, 2018) or from previous reports (Song et al., 2005; Röder et al., 1998; Somers et al. 2004; Sourdille et al., 2003; Pestsova et al., 2000; Guyomarc’h et al., 2002; Eujayl et al., 2002; Yu et al., 2004; Barkley et al., 2006).  ^b^Symbol “/” indicates the marker mapped to two or more chromosomes in wheat or *Ae. markgrafii*. | | | | |
